# Pan-cancer patterns of cuproptosis markers reveal biologically and clinically relevant cancer subtypes

**DOI:** 10.1186/s40364-022-00446-5

**Published:** 2023-01-31

**Authors:** Fanqin Bu, Xiangji Li, Yu Zhao, Liyi Bai, Shutian Zhang, Li Min

**Affiliations:** Department of Gastroenterology, Beijing Friendship Hospital, Capital Medical University, National Clinical Research Center for Digestive Disease, Beijing Digestive Disease Center, Beijing Key Laboratory for Precancerous Lesion of Digestive Disease, Beijing, 100050 P. R. China

**Keywords:** Cuproptosis, Tumor microenvironment, Pan-cancer

## Abstract

Cuproptosis is a newly discovered type of cell death triggered by copper accumulation. Here we exhibited the genetic profiles of 10 cuproptosis-associated genes (CuAGs) across 21 cancer types. Only 8.0% (627/7839) of tumors possessed at least 1 mutation on CuAGs, while the copy number amplifications or deletions on the alleles of CuAGs were ubiquitous. Generally, the expression of CuAGs showed heterogeneity across cancer types and the expression of CuAGs showed different correlations with MSI, TMB, immune and stromal features in different cancer types. Therefore, CuAGs were ubiquitously and heterogeneously dysregulated in pan-cancer. With a Non-negative Matrix Factorization method, we divided patients of each cancer type into cuproptosis-based subtypes, which showed a close but heterogeneous correlation with different biological and clinical features. Accordingly, we summarized all cancer types into four categories. The cancers in which cuproptosis subtypes correlated with MSI and TMB were annotated as Genomic disturbed. Those correlated with stromal scores were categorized as Stromal remolded. The others only associated with immune infiltration were labeled as Immune inhibited. A minor fraction of cancers not correlated with any biological indicators were marked as Cuproptosis inert. Together, we provided a pan-cancer overview of cuproptosis markers which revealed biologically and clinically relevant cancer subtypes in different cancers.

**Supplementary Information:**

The online version contains supplementary material available at 10.1186/s40364-022-00446-5.

## To the editor

Cuproptosis, a unique type of cell death triggered by copper accumulation, was newly characterized [1]. The inner mechanism of cuproptosis involved mitochondrial protein lipoylation, tricarboxylic acid cycle, and reactive oxygen species generation, which were strongly associated with tumor microenvironment (TME) homeostasis [1–3]. However, whether the cuproptosis-associated genes (CuAGs) were associated with cancer progression and the TME features were still unknown. In this study, we collected CuAGs which encoded the components of the lipoic acid pathway (FDX1, LIAS, LIPT1, DLD) and the pyruvate dehydrogenase complex (DLAT, PDHA1, PDHB, MTF1, GLS, CDKN2A), including 3 negative regulators (*i.e.*, MTF1, GLS, CDKN2A) and other 7 positive regulators. Then we employed gene expression data of those 10 CuAGs from the TCGA database to reveal the cuproptosis-associated distinct regulatory patterns in different cancers.

**Fig. 1:**
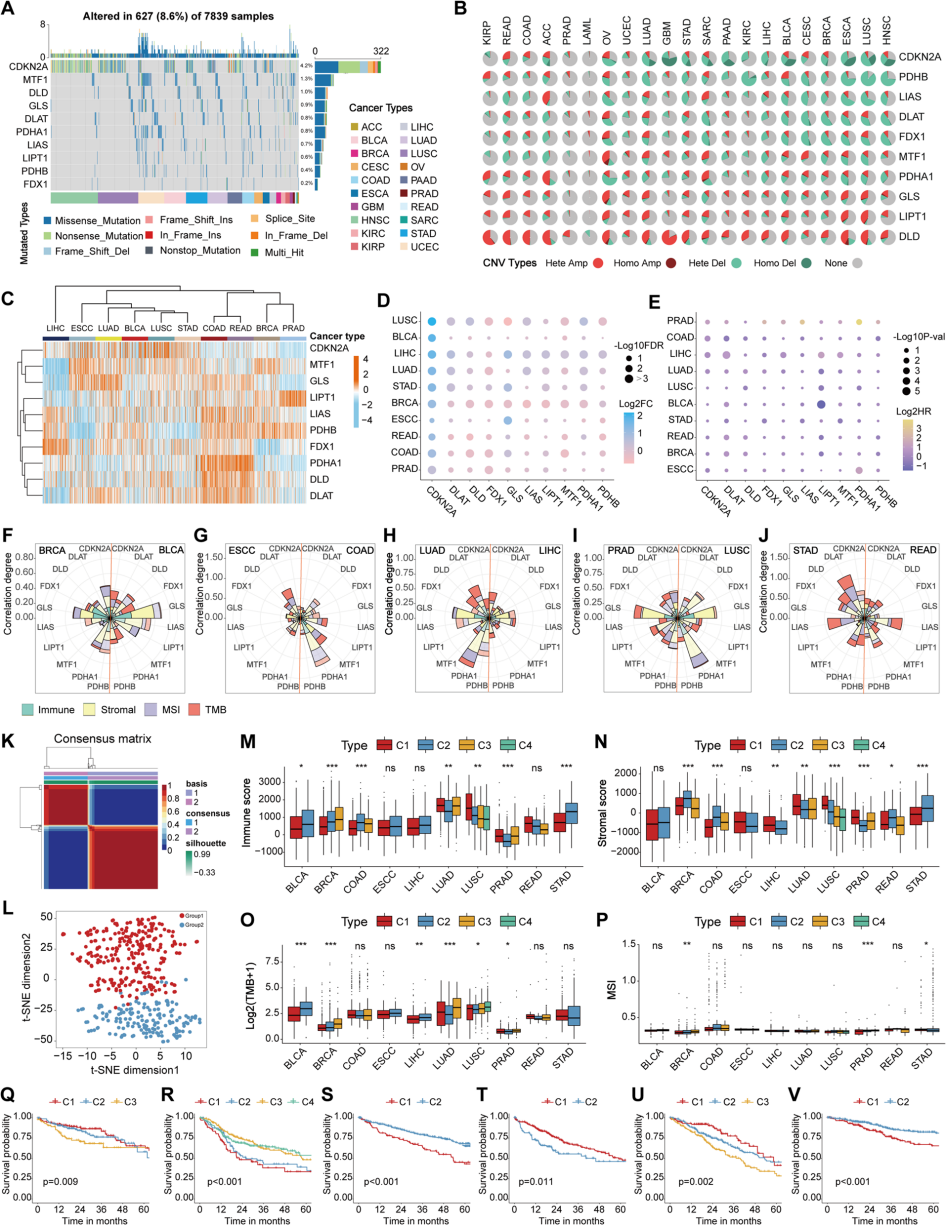
Multi-omics features of cuproptosis markers in pan-cancer. **A**. Somatic mutation status of cuproptosis markers in 20 cancer types visualized via waterfall plot. Top bar plot showing the mutated gene counts of cuproptosis markers in each sample, the numbers between waterfall plot and mutated types showing the mutated frequency of each cuproptosis markers. **B**. Pie plot showing the copy number variations of cuproptosis markers in 21 cancers. **C**. Expressional difference of cuproptosis markers among 10 cancer types visualized via heatmap, 100 samples were randomly selected for each cancer. **D**. Differential expression of cuproptosis markers in different cancers compared with adjacent normal tissues showed by dot plot. **E**. Dot plot showing the prognostic value of cuproptosis markers in different cancers. **F**-**J**. Petal graph showing the multi-features correlation degrees of cuproptosis markers in 10 cancers, covering immune score, stromal score, MSI and TMB. The cancer types are shown in the upper left or upper right corner, respectively. The length of the petals indicated the correlation degrees, opaque petals indicated positive correlations and translucent petals indicated negative correlations. **K**. NMF clustering results via cuproptosis markers for dimensionality reduction in BLCA patients. **L**. Validation of the clustering result of BLCA patients showed by two-dimensional t-SNE plot. **M**. Boxplot showing the intergroup comparing results of immune scores in 10 cancers. Median-lines in each box represented median value, different asterisks indicated the results of *p-value* (*P < 0.05; **P < 0.01; ***P < 0.001) **N**. Boxplot showing the intergroup comparing results of stromal scores in 10 cancers. Median-lines in each box represented median value, different asterisks indicated the results of *p-value* (*P < 0.05; **P < 0.01; ***P < 0.001). **O** Boxplot showing the intergroup comparing results of TMB in 10 cancers. Median-lines in each box represented median value, different asterisks indicated the results of *p-value* (*P < 0.05; **P < 0.01; ***P < 0.001). **P** Boxplot showing the intergroup comparing results of MSI in 10 cancers. Median-lines in each box represented median value, different asterisks indicated the results of *p-value* (*P < 0.05; **P < 0.01; ***P < 0.001). **Q**-**V**. Survival curves revealed the prognostic difference between different subgroups in COAD (**Q**), HNSC (**R**), KIRC (**S**), LIHC (**T**), LUAD (**U**), and UCEC (**V**) separately

Here we found that the overall somatic mutational frequency of the 10 CuAGs was at a moderate level (8.0%, 627/7839), and CDKN2A exhibited the highest somatic mutation rates (Fig. 1A, Additional file 1: Figure S1A). Compared with the mutation status, the CNV alterations are much more prevalent, and the CNV status of each CuAG was comparably consistent among different cancer types (Fig. 1B). The expression of CuAGs exhibited high heterogeneity among different cancers (Fig. 1C), among which CDKN2A was overexpressed in most of the cancers, even with a deletion in CNV (Fig. 1D, Additional file 1: Figure S1B-K). Generally, all of the CuAGs showed expressional dysregulation and prognostic relevance (Fig. 1D-E), among which DLAT was the most widely applicable prognostic CuAG that correlated with the prognosis of 7 different cancers (Additional file 2: Table S1). KIRC was the cancer type most affected by CuAGs in prognosis, in which 7/10 CuAGs were prognostic (Additional file 2: Table S 1). Moreover, CDKN2A, DLAT, GLS, LIAS, and PDHB were also identified as potential chemotherapy response predictors (Additional file 1: Figure S2).

Then the correlations between the CuAGs and biological features of different cancers were evaluated. Most CuAGs exhibited close and consistent associations with the immune score and the stromal score (Fig. 1F-J, Additional file 2: Table S2 and S3). The 7 positive cuproptosis regulators exhibited negative correlations with both immune and stromal scores in the majority of the cancer types, while the 3 negative cuproptosis regulators shared positive associations with TME scores (Additional file 1: Figure S3 and S4). Comparably, the regulation of CuAGs on TMB and MSI was heterogeneous (Additional file 1: Figure S5 and S6, Additional file 2: Table S4 and S5), suggesting that cuproptosis might affect genomic stability only in some cancer types.

To evaluate the overall correlation between cuproptosis and different biological features in various cancers, we employed the non-negative matrix (NMF) factorization to classify patients based on all CuAGs [4]. For each cancer, patients were assorted into 2-4 subgroups (Fig. 1K, Additional file 1: Figure S7). The 2D t-SNE plot verified the mathematical validity of the unsupervised clustering results by NMF (Fig. 1L, Additional file 1: Figure S8). In most cancer types, the NMF subgroups exhibited a strong correlation with immune (15/21) and stromal scores (11/21) [5] (Fig. 1M-N, Additional file 1: Figure S9A-B). Correspondently, correlations between genomic stability indicators [6, 7] and NMF subgroups were only identified in a small portion of cancers (9/21 for TMB score, Fig. 1O, Additional file 1: Figure S9C; 6/21 for MSI scores, Fig. 1P, Additional file 1: Figure S9D). Additionally, in most cancer types, subgroups showed close association with cuproptosis-related biological processes, such as hypoxia and reactive oxygen species (ROS) pathways [8, 9] (Additional file 1: Figure S10). Meanwhile, in COAD, HNSC, KIRC, LIHC, LUAD, and UCEC, different NMF subgroups also exhibited distinct overall survivals (Fig. 1Q-V, Additional file 1: Figure S11).

**Fig. 2:**
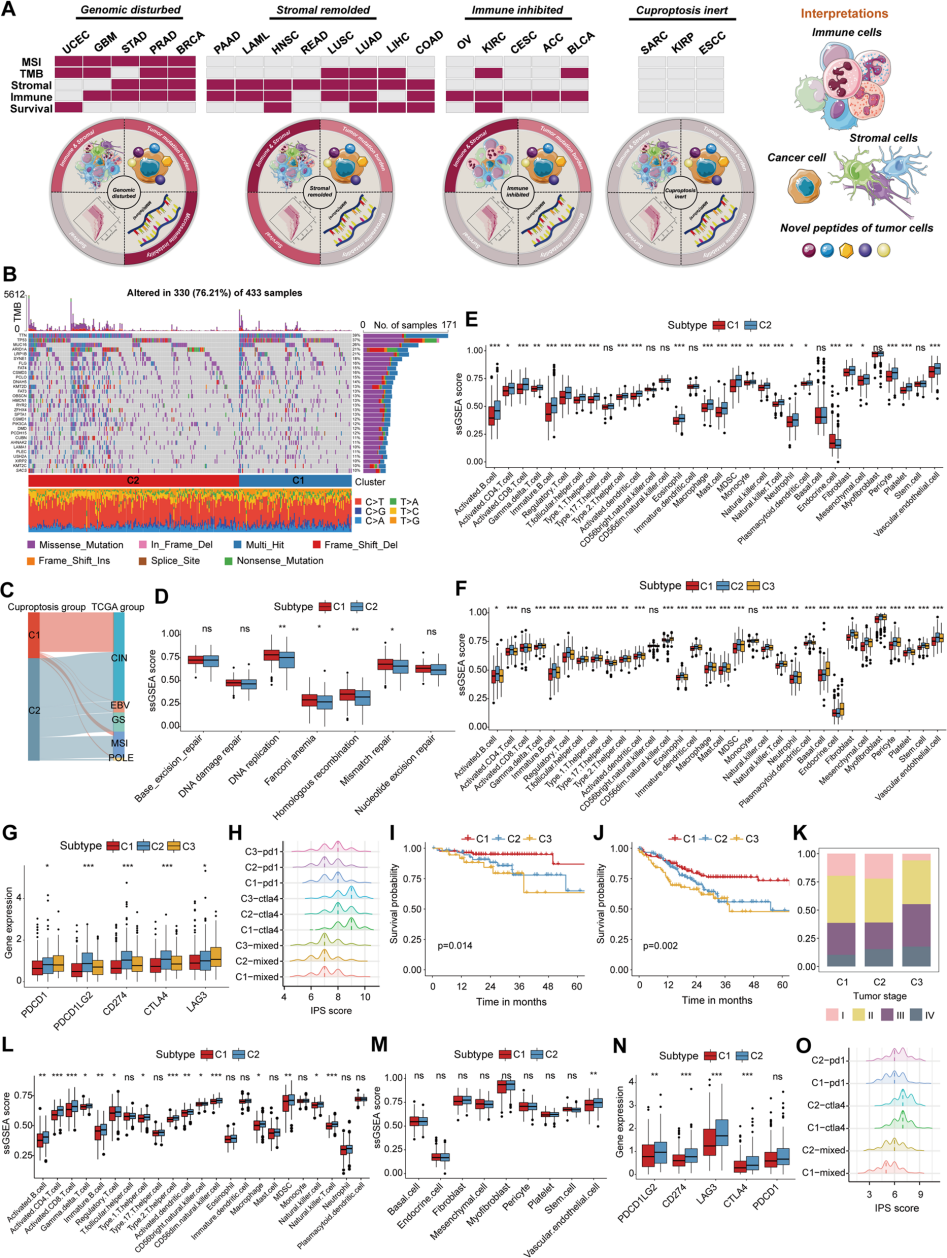
Classification of cancers based on regulatory patterns of cuproptosis genes. **A** Diagram showing the cuproptosis regulating patterns in different cancers and the classification of cancers with distinct cuproptosis subtypes. **B** The waterfall plot depicting the overall mutation status of top30 mutated genes in different STAD subgroups. The upper box indicates the TMB of each sample, the right columns show the mutated types of each gene, the numbers between the waterfall plot and right columns show the mutated frequency of each gene, and the stacked bars below show the fraction of conversions in each sample. **C** Sankey diagram depicting the sample distributions between cuproptosis groups and traditional TCGA STAD classifications. **D** Boxplot showing the intergroup differences in DNA repairing scores in STAD patients. Median lines in each box represented the median value, different asterisks indicated the results of *p-value* (*P < 0.05; **P < 0.01). **E** Boxplot showing the intergroup differences in the infiltrations of TME-related cells in STAD patients. Median lines in each box represented the median value, different asterisks indicated the results of *p-value* (*P < 0.05; **P < 0.01; ***P < 0.001). **F** Boxplot showing the intergroup differences in the infiltrations of TME-related cells in COAD patients. Median lines in each box represented the median value, different asterisks indicated the results of *p-value* (*P < 0.05; **P < 0.01; ***P < 0.001). **G** The expression profile of immune checkpoint genes in different COAD subgroups. Median lines in each box represented the median value, different asterisks indicated the results of *p-value* (*P < 0.05; ***P < 0.001). **H** The hill plot exhibits the distributions of IPS values on anti-PD1 and anti-CTLA4 immunotherapy between different subgroups of COAD patients. Dotted lines in each hill show the median IPS value. **I **Survival curve showing the disease-free survival (DFS) difference among different cuproptosis groups in COAD. **J** Survival curve showing the progression-free survival (PFS) difference among different cuproptosis groups in COAD. **K** The histogram showing the COAD sample distributions in different tumor stages. **L** Boxplot showing the intergroup differences in the infiltrations of immune cells in OV patients. Median lines in each box represented the median value, different asterisks indicated the results of *p-value* (*P < 0.05; **P < 0.01; ***P < 0.001). **M** Boxplot showing the intergroup differences in the infiltrations of stromal cells in OV patients. Median lines in each box represented the median value, the asterisks indicated the results of *p-value* (**P < 0.01). **N** The expression profile of immune checkpoint genes in different OV subgroups. Median lines in each box represented the median value, different asterisks indicated the results of *p-value* (**P < 0.01; ***P < 0.001). **O** The hill plot exhibits the distributions of IPS value on anti-PD1 and anti-CTLA4 immunotherapy between different subgroups of OV patients. Dotted lines in each hill show the median IPS value

Since CuAGs showed a heterogeneous correlation with different biological and clinical features, we further summarized all cancer types into four categories: Genomic disturbed, Stromal remolded, Immune inhibited, and Cuproptosis inert (Fig. 2A, Additional file 2: Table S6). The Genomic disturbed category refers to the cancers most dramatically affected by CuAGs, and their NMF subgroups were generally associated with both genomic instability indicators and tumor microenvironment (e.g., STAD, Fig. 2B-E). The Stromal remolded category refers to the cancers in which the NMF subgroups correlated with the TME-related features but not the genomic-stability ones. Moreover, the Stromal remolded category was also characterized by closely associated with prognosis (e.g., COAD, Fig. 2F-K, Additional file 1: Figure S12). The Immune inhibited category refers to the cancers in which the NMF subtype exhibited correlations mainly with immune scores. We believe that those cancers were only slightly affected by CuAGs, in which cuproptosis only affected the immune microenvironment (e.g., OV, Fig. 2L-O, Additional file 1: Figure S13). The Cuproptosis inert category, which was annotated for the cancers barely affected by cuproptosis, was not associated with any biological and clinical features (e.g., SARC, Additional file 1: Figure S14).

In conclusion, we demonstrated the widespread genetic dysregulation and distinct biological relevance of cuproptosis regulators in pan-cancer. Moreover, we provided a pan-cancer overview of cuproptosis regulating patterns which revealed biologically and clinically relevant cancer subtypes.

## Cancers with abbreviations

ACC, adrenocortical carcinoma; BLCA, bladder urothelial carcinoma; BRCA, breast cancer; CESC, cervical squamous cell carcinoma, and endocervical adenocarcinoma; COAD, colon adenocarcinoma; ESCC, esophageal carcinoma; GBM, glioblastoma multiforme; HNSC, head, and neck squamous carcinoma; KIRC, kidney renal clear cell carcinoma; KIRP, kidney renal papillary cell carcinoma; LAML, acute myeloid leukemia; LIHC, liver hepatocellular carcinoma; LUAD, lung adenocarcinoma; LUSC, lung squamous cell carcinoma; OV, ovarian serous cystadenocarcinoma; PAAD, pancreatic adenocarcinoma; PRAD, prostate adenocarcinoma; READ, rectum adenocarcinoma; SARC, sarcoma; STAD, stomach adenocarcinoma; UCEC, uterine corpus endometrial carcinoma.

## Supplementary Information

**Additional file 1:** **Fig. S1.** Somatic mutation and expression of cuproptosis markers in cancers. **Fig. S2.** GDSC data revealing the sensitivity to different drugs of cuproptosis markers. **Fig. S3.** The correlation plot exhibiting the correlations between cuproptosis markers and immune scores in 21 TCGA cancers. **Fig. S4.** The correlation plot exhibiting the correlations between cuproptosis markers and stromal scores in 21 TCGA cancers. **Fig. S5.** The correlation plot exhibiting the correlations between cuproptosis markers and TMB in 21 TCGA cancers. **Fig. S6.** The correlation plot exhibiting the correlations between cuproptosis markers and MSI in 21 TCGA cancers. **Fig. S7.** NMF clustering of different cancers based on cuproptosis markers. **Fig. S8.** Visualization of NMF subtypes via two-dimensional t-SNE. **Fig. S9.** Differences in biological features between different NMF subgroups in pan-cancer. **Fig. S10.** Boxplots showing the hallmark scores of Hypoxia and Reactive oxygen species among different subgroups. **Fig. S11.** Prognosis analysis comparing different NMF subgroups in pan-cancer. **Fig. S12.** Analysis on different biological features between different NMF subgroups in COAD patients. **Fig. S13.** Analysis on different biological features between different NMF subgroups in OV patients. **Fig. S14.** Analysis on different biological features between different NMF subgroups in SARC patients.

**Additional file 2:** **Table S1.** Prognostic value of CuAGs in pan-cancer. **Table S2.** Pearson correlation between CuAGs expressions and immune scores in pan-cancer. **Table S3.** Pearson correlation between CuAGs expressions and stromal scores in pan-cancer. **Table S4.** Pearson correlation between CuAGs expressions and TMB in pan-cancer. **Table S5.** Pearson correlation between CuAGs expressions and MSI in pan-cancer. **Table S6.** An ultimate cancer classification system based on distinct regulatory patterns of CuAGs. **Table S7.** The gene lists used for marking DNA repairing-related pathways. **Table S8.** The gene lists used for marking TME-related cells. **Table S9.** The gene lists used for marking TME-related pathways.

**Additional file 3** .

## Data Availability

All sequencing data were public available datasets (TCGA https://portal.gdc.cancer.gov/cart). All other data supporting the conclusions of this article are presented within the article and its supplementary files.

## Abbreviations

CNV: Copy number variations
CuAG: Cuproptosis associated gene
DFS: Disease-free survival
GS: Genome stable
GSEA: Gene set enrichment analysis
IPS: Immunophenoscore
MSI: Microsatellite instability
NMF: Non-negative matrix factorization
OS: Overall survival
PFS: Progression-free survival
ROS: Reactive oxygen species
ssGSEA: Single-sample gene-set enrichment analysis
TCGA: The Cancer Genome Atlas
TME: Tumor microenvironment
TMB: Tumor mutation burden
