# Supplementary material for: Pan-cancer patterns of cuproptosis markers reveal biologically and clinically relevant cancer subtypes
Source: Biomark Res. 2023 Jan 31;11:13. doi: 10.1186/s40364-022-00446-5 (PMC9887831; doi:10.1186/s40364-022-00446-5)
Supplement: Supplementary file 1 — Additional file 1: Fig. S1. Somatic mutation and expression of cuproptosis markers in cancers. Fig. S2. GDSC data revealing the sensitivity to different drugs of cuproptosis markers. Fig. S3. The correlation plot exhibiting the correlations between cuproptosis markers and immune scores in 21 TCGA cancers. Fig. S4. The correlation plot exhibiting the correlations between cuproptosis markers and stromal scores in 21 TCGA cancers. Fig. S5. The correlation plot exhibiting the correlations between cuproptosis markers and TMB in 21 TCGA cancers. Fig. S6. The correlation plot exhibiting the correlations between cuproptosis markers and MSI in 21 TCGA cancers. Fig. S7. NMF clustering of different cancers based on cuproptosis markers. Fig. S8. Visualization of NMF subtypes via two-dimensional t-SNE. Fig. S9. Differences in biological features between different NMF subgroups in pan-cancer. Fig. S10. Boxplots showing the hallmark scores of Hypoxia and Reactive oxygen species among different subgroups. Fig. S11. Prognosis analysis comparing different NMF subgroups in pan-cancer. Fig. S12. Analysis on different biological features between different NMF subgroups in COAD patients. Fig. S13. Analysis on different biological features between different NMF subgroups in OV patients. Fig. S14. Analysis on different biological features between different NMF subgroups in SARC patients. [file 40364_2022_446_MOESM1_ESM.docx]

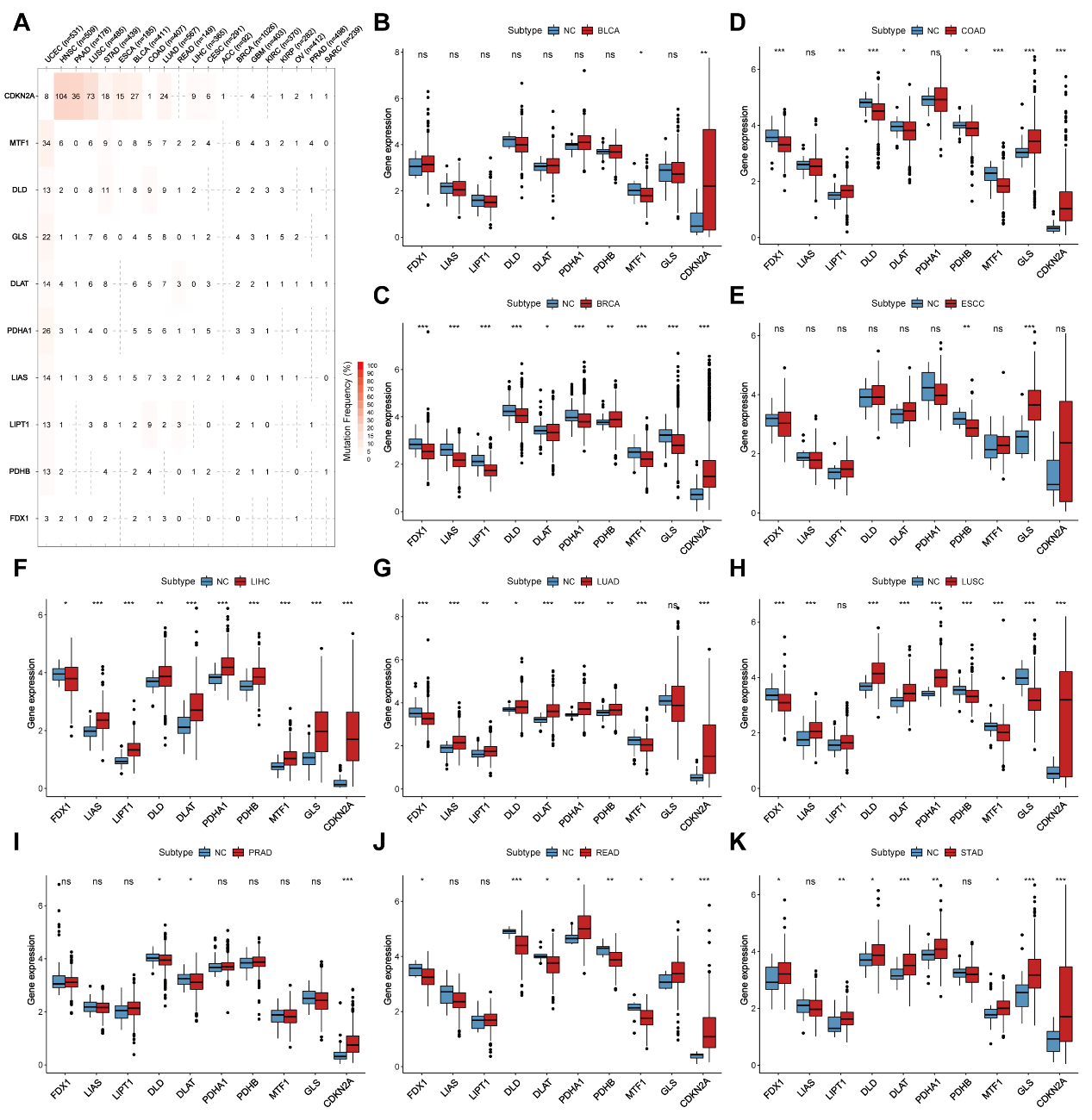


**Figure S1. Somatic mutation and expression of cuproptosis markers in cancers**

A. Heatmap showing the somatic mutation of cuproptosis markers in 20 cancers, the numbers in the box represent mutated samples.

B-K. Boxplots showing the expression of cuproptosis markers in 10 cancers and corresponding adjacent tissues.

The median line in each box represented the median value, different asterisks indicated the results of *p-value* (**P* < 0.05; ***P* < 0.01; ****P* < 0.001).


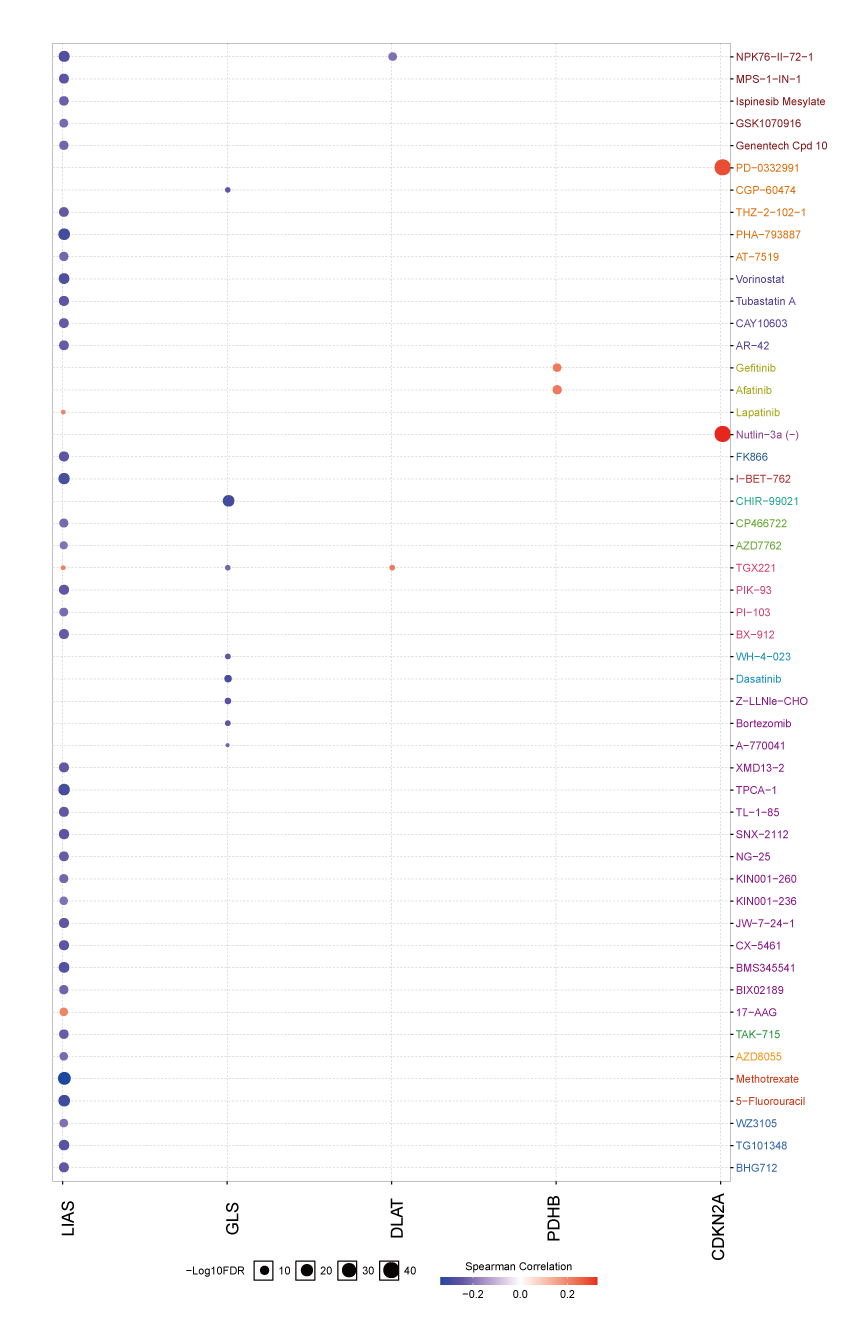


**Figure S2. GDSC data revealing the sensitivity to different drugs of cuproptosis markers**


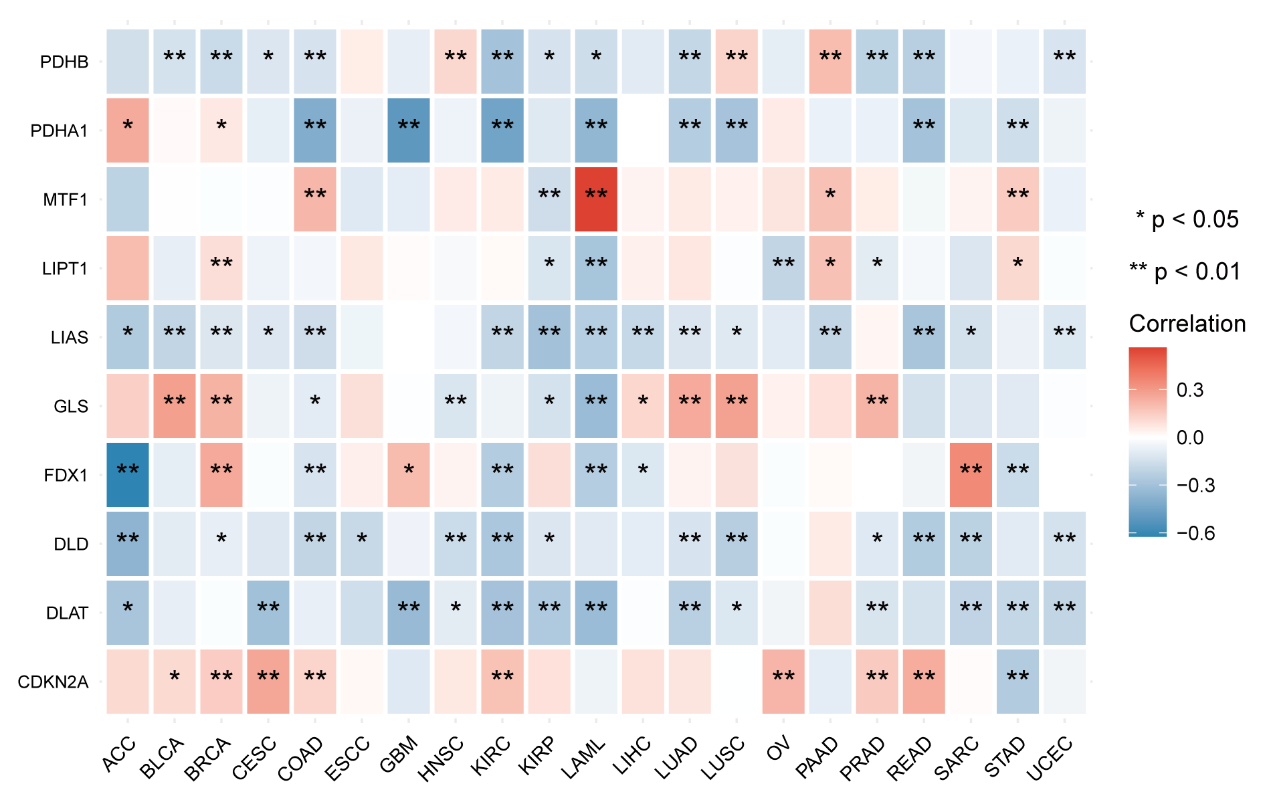


**Figure S3. The correlation plot exhibiting the correlations between cuproptosis markers and immune scores in 21 TCGA cancers.**


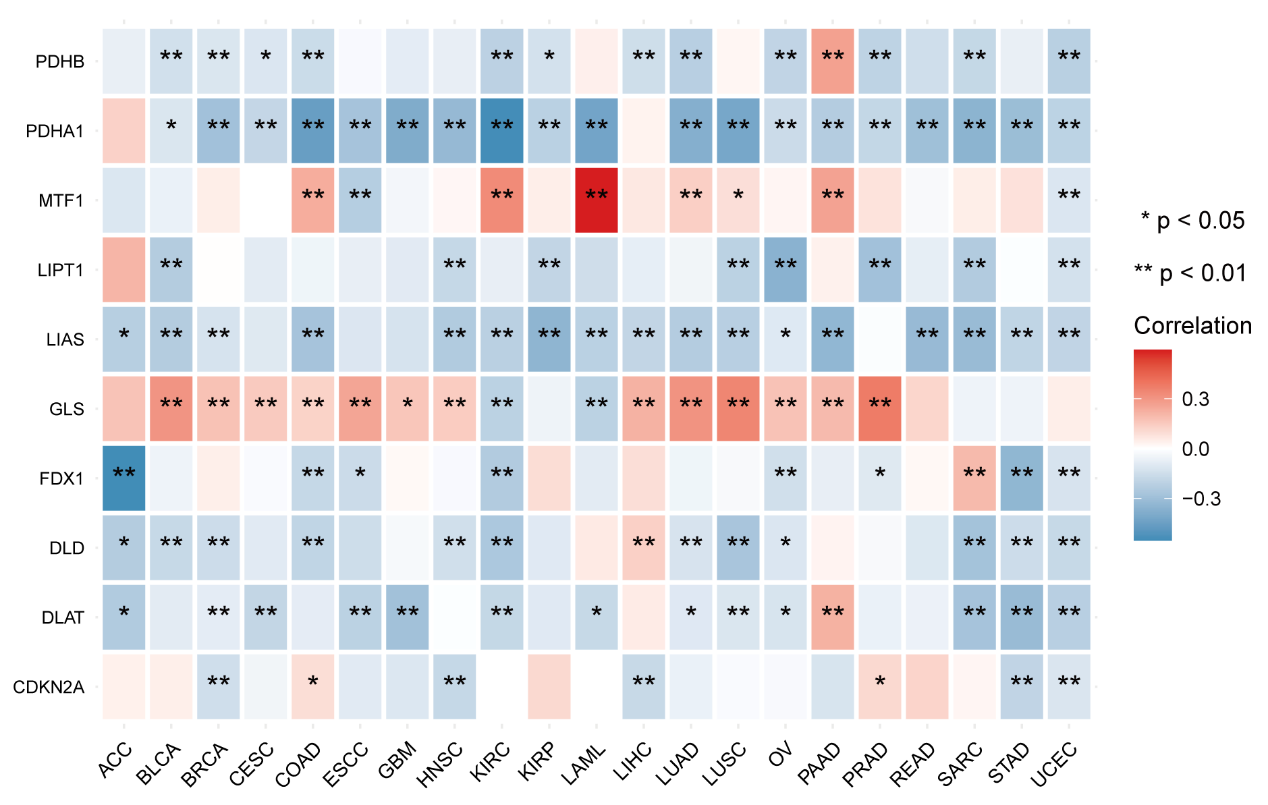


**Figure S4. The correlation plot exhibiting the correlations between cuproptosis markers and stromal scores in 21 TCGA cancers.**


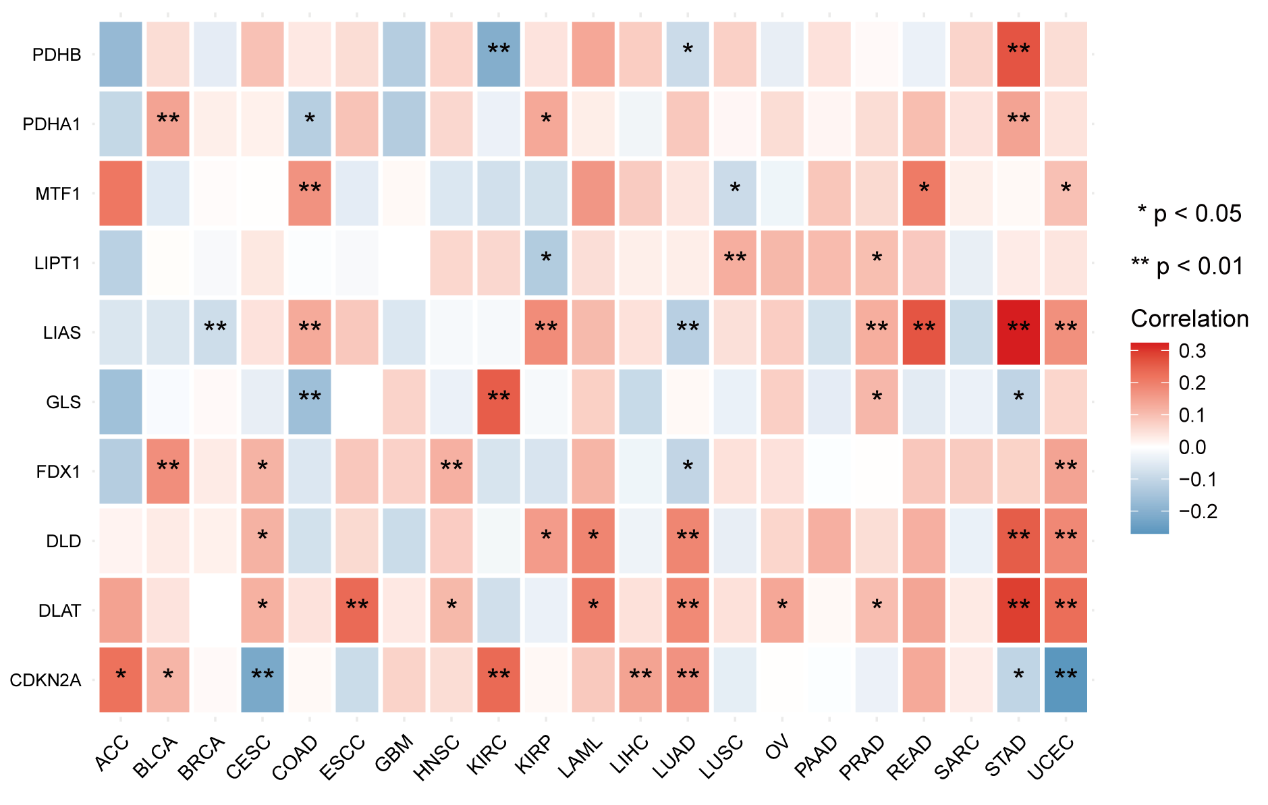


**Figure S5. The correlation plot exhibiting the correlations between cuproptosis markers and TMB in 21 TCGA cancers.**


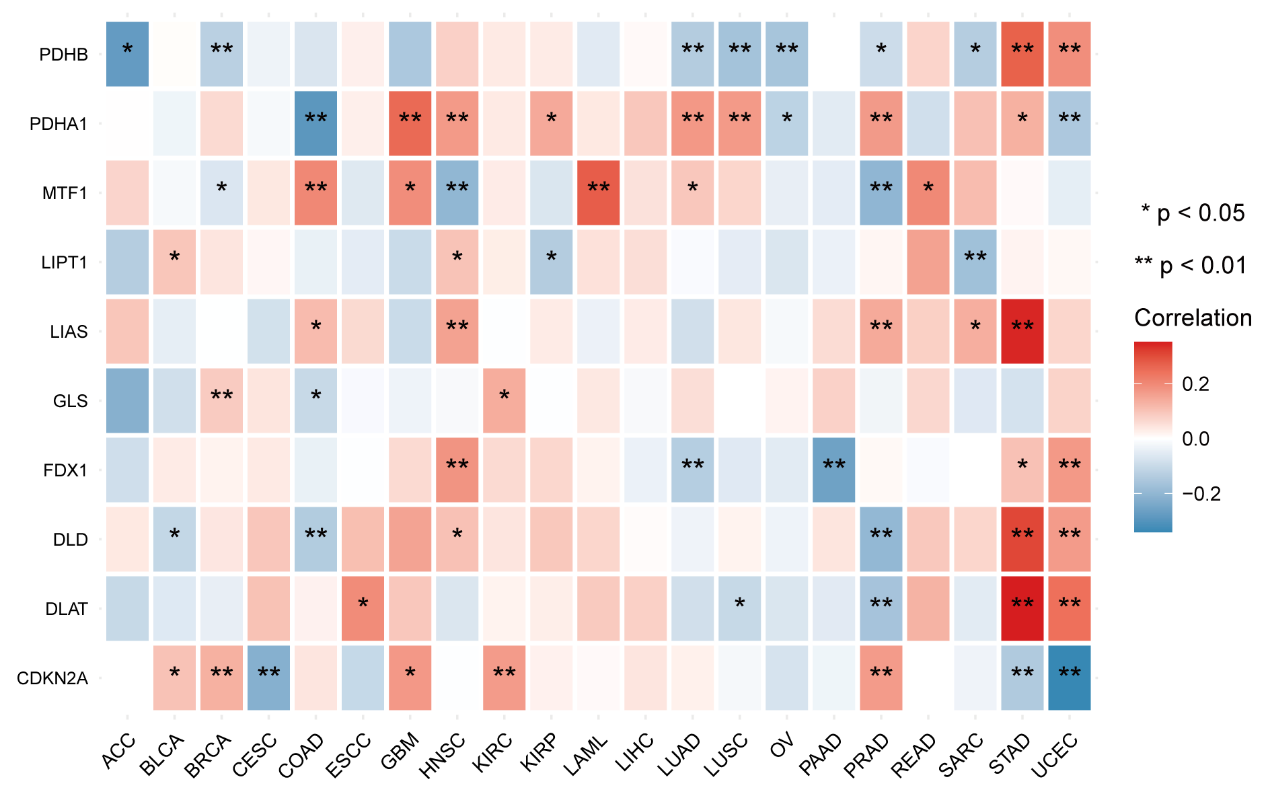


**Figure S6. The correlation plot exhibiting the correlations between cuproptosis markers and MSI in 21 TCGA cancers.**


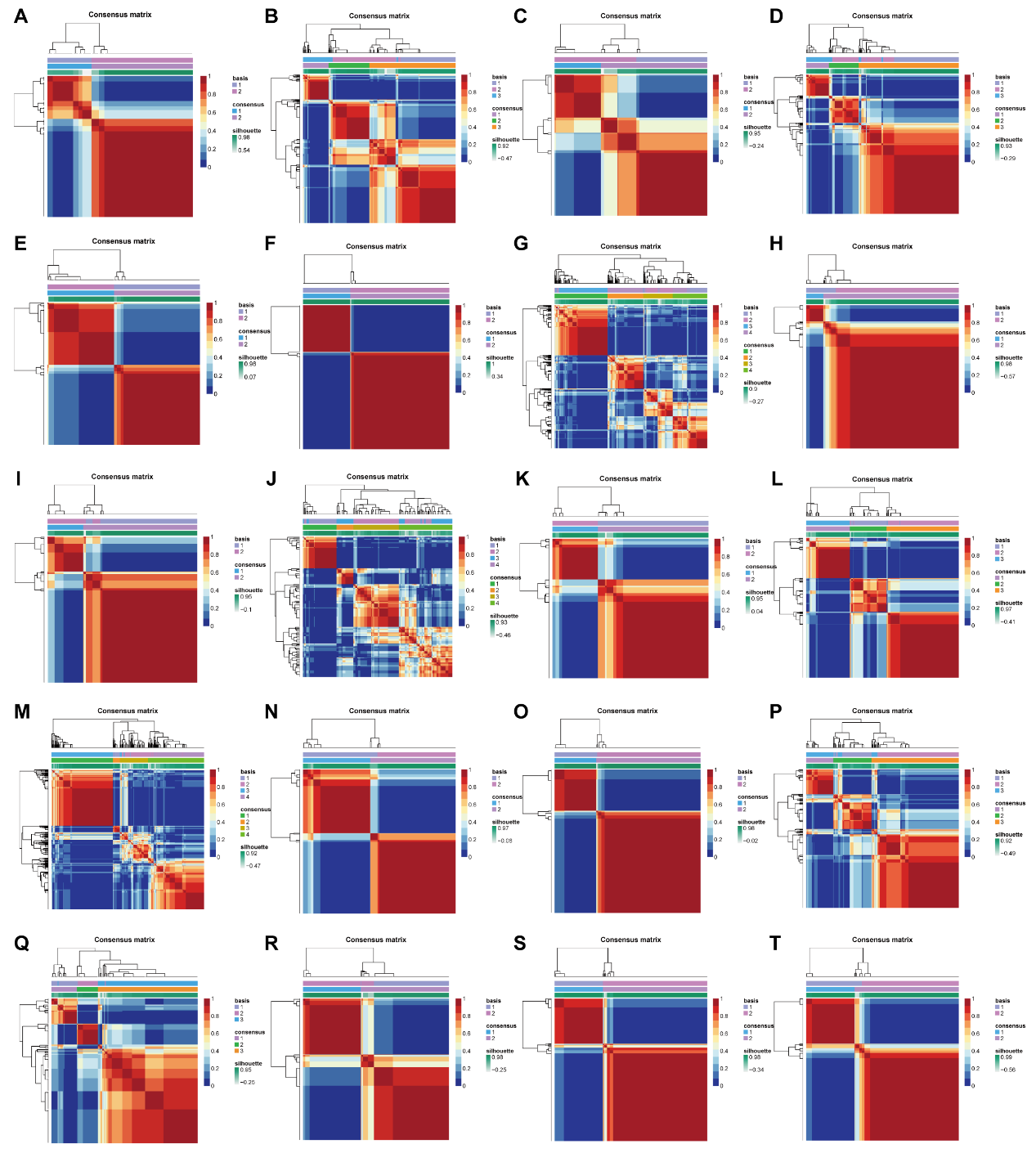


**Figure S7. NMF clustering of different cancers based on cuproptosis markers**

Clustering results of ACC (A), BRCA (B), CESC (C), COAD (D), ESCC (E), GBM (F), HNSC (G), KIRC (H), KIRP (I), LAML (J), LIHC (K), LUAD (L), LUSC (M), OV (N), PAAD (O), PRAD (P), READ (Q), SARC (R), STAD (S), and UCEC (T).


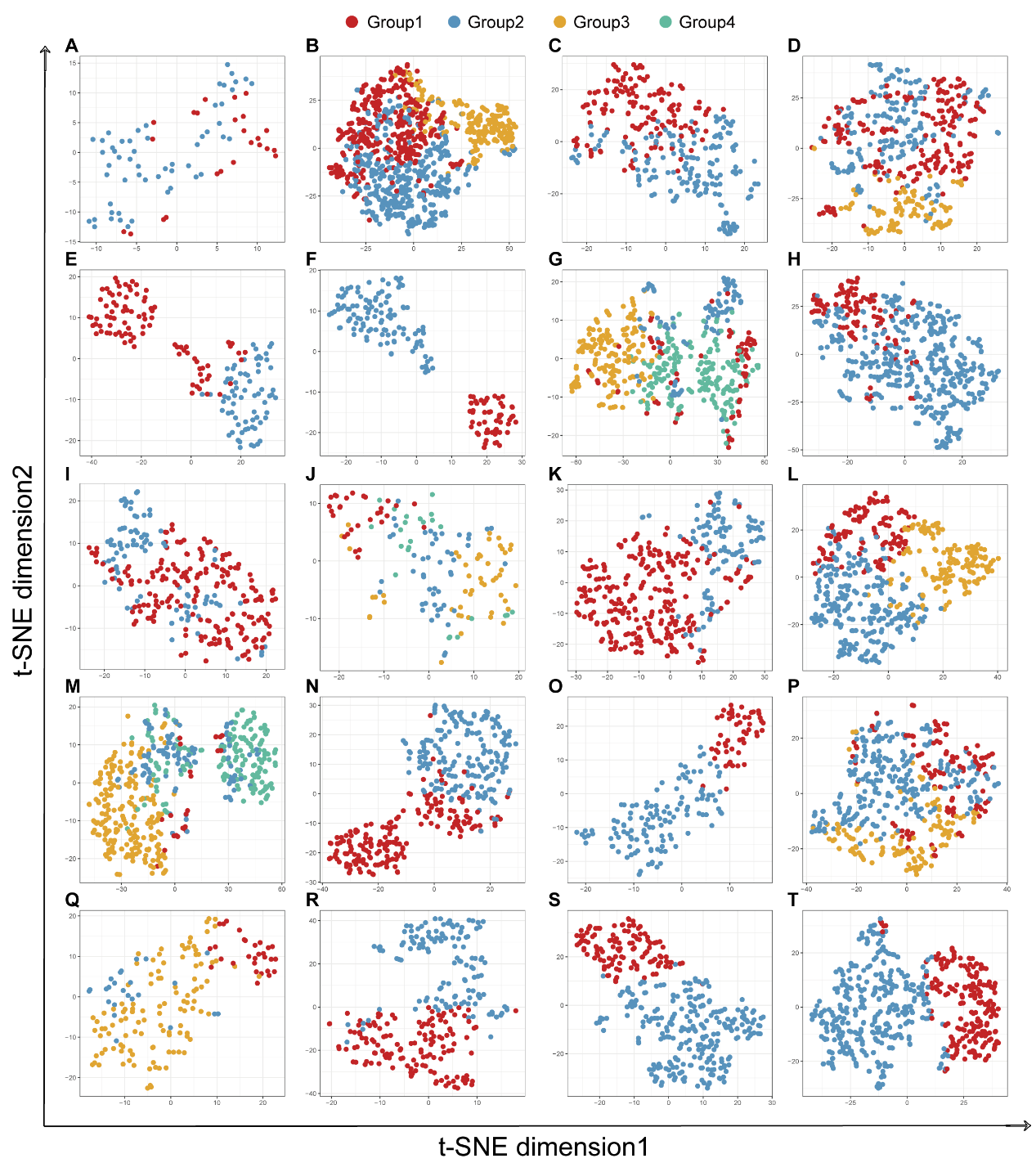


**Figure S8. Visualization of NMF subtypes via two-dimensional t-SNE**

T-SNE plots of ACC (A), BRCA (B), CESC (C), COAD (D), ESCC (E), GBM (F), HNSC (G), KIRC (H), KIRP (I), LAML (J), LIHC (K), LUAD (L), LUSC (M), OV (N), PAAD (O), PRAD (P), READ (Q), SARC (R), STAD (S), and UCEC (T).


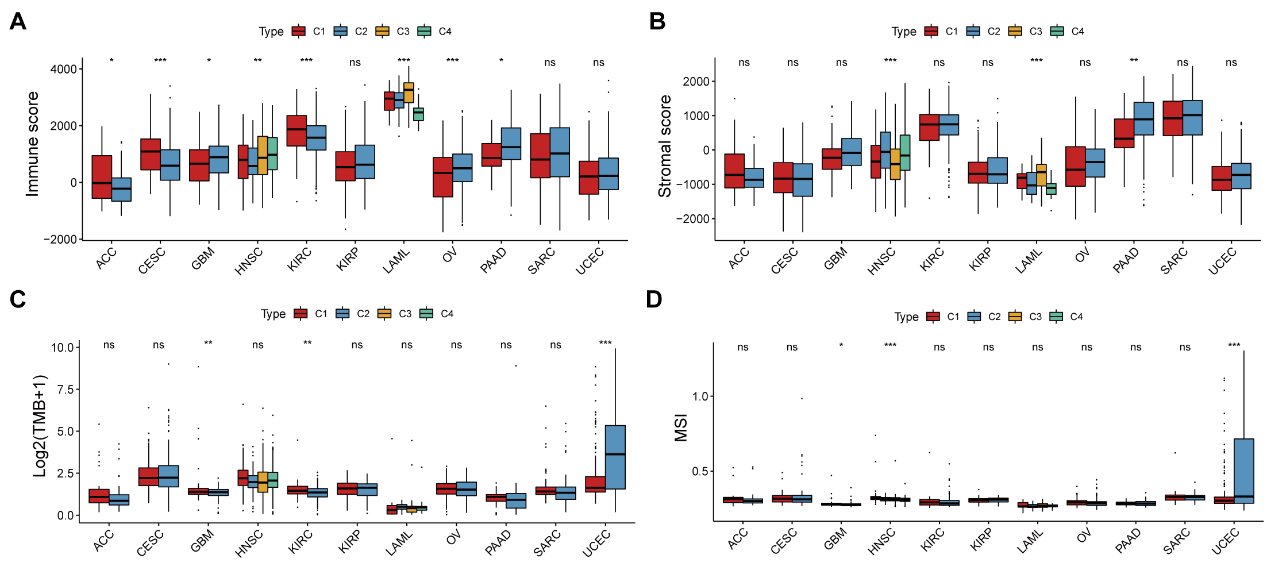


**Figure S9. Differences in biological features between different NMF subgroups in pan-cancer**

A. Boxplot showing the immune scores in 11 cancers.

B. Boxplot showing the stromal scores in 11 cancers.

C. Boxplot showing the TMB in 11 cancers.

D. Boxplot showing the MSI in 11 cancers.

The median-lines in each box represented median value, different asterisks indicated the results of *p-value* (**P* < 0.05; ***P* < 0.01; ****P* < 0.001).


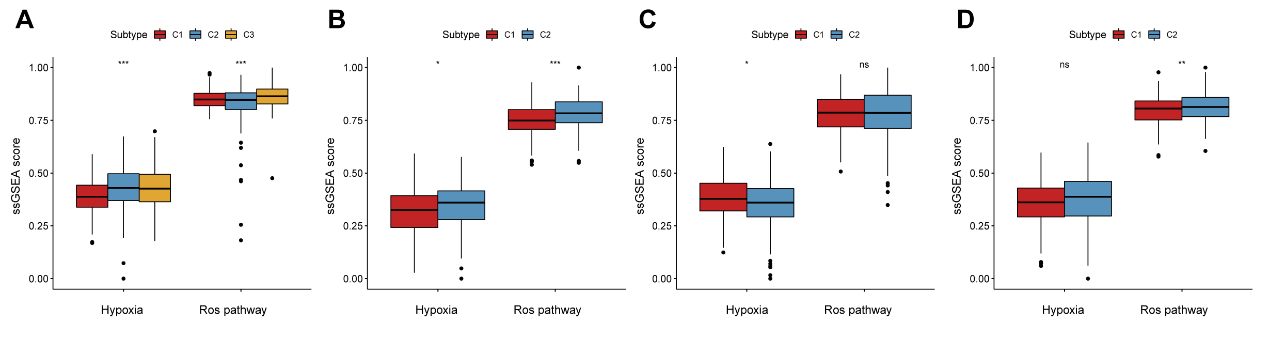


**Figure S10. Boxplots showing the hallmark scores of Hypoxia and Reactive oxygen species among different subgroups**

Results in COAD (A), OV (B), SARC (C), and STAD (D). The median line in each box represented the median value, different asterisks indicated the results of *p-value* (**P* < 0.05; ***P* < 0.01; ****P* < 0.001).


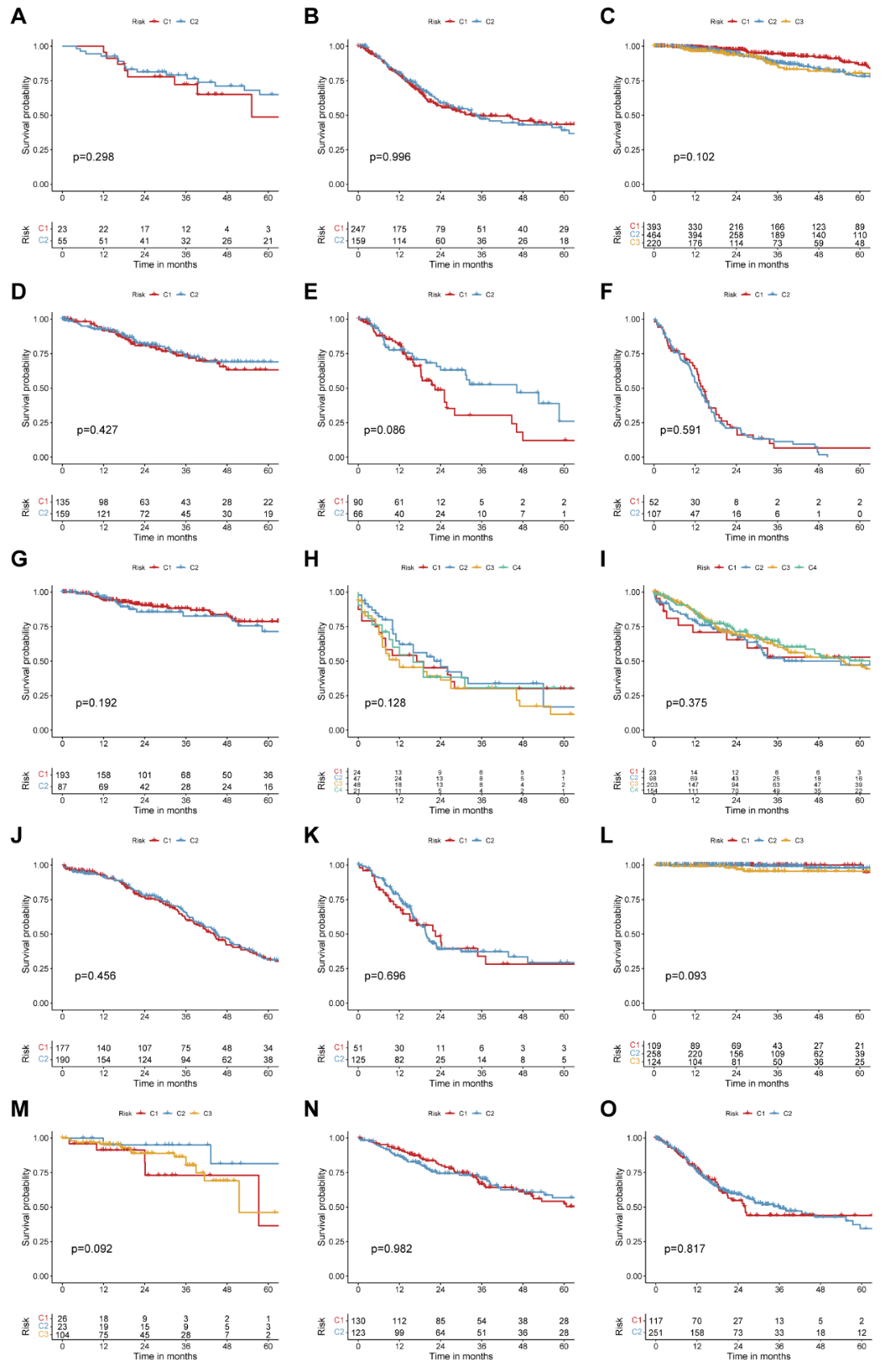


**Figure S11. Prognosis analysis comparing different NMF subgroups in pan-cancer**

A-O. The differences in overall survival showing by Kaplan-Meier curves in ACC (A), BLCA (B), BRCA (C), CESC (D), ESCC (E), GBM (F), KIRP (G), LAML (H), LUSC (I), OV (J), PAAD (K), PRAD (L), READ (M), SARC (N), STAD (O).


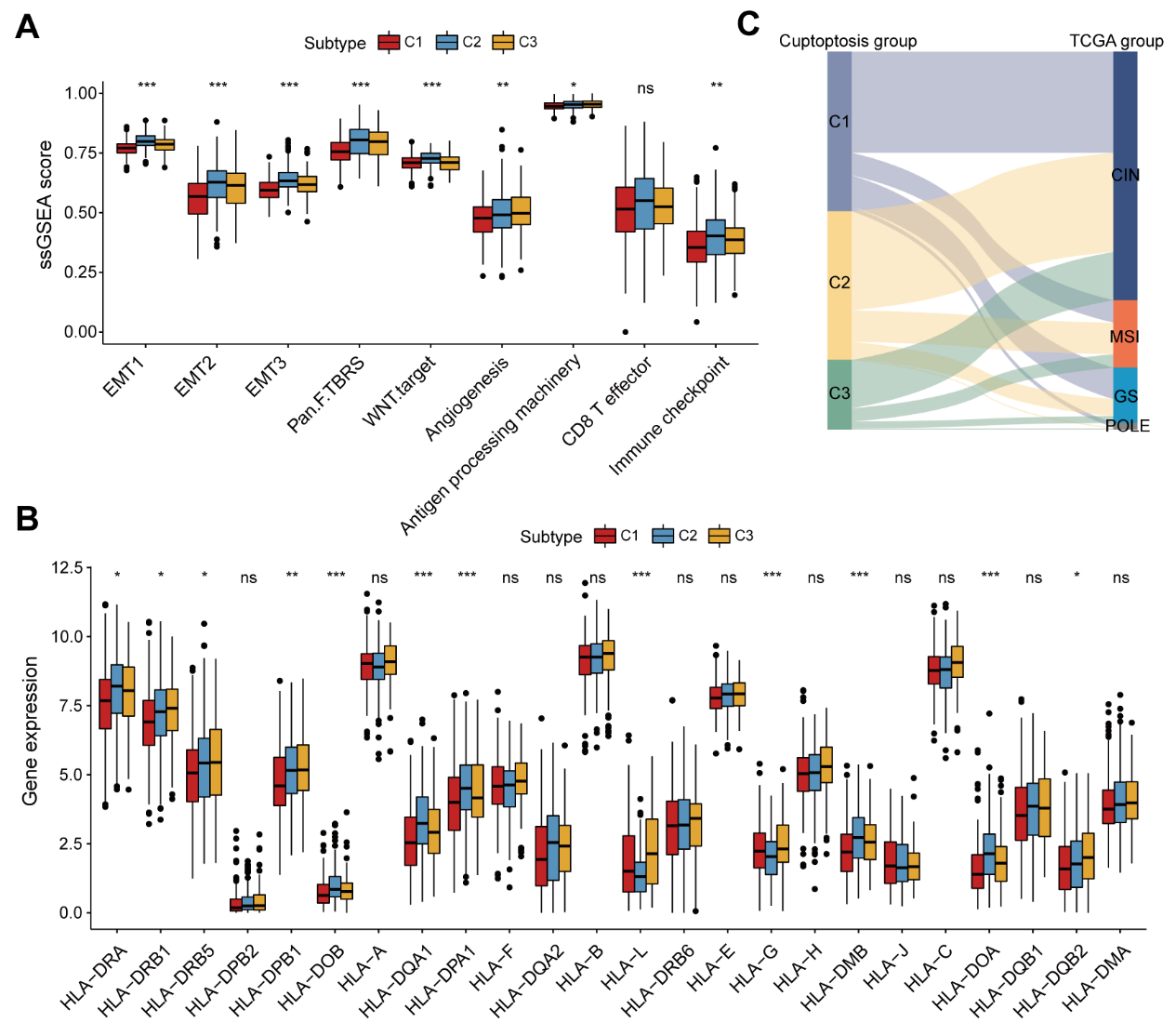


**Figure S12. Analysis on different biological features between different NMF subgroups in COAD patients**

A. Boxplot showing the TME-related pathways in COAD patients.

B. Boxplot showing the HLA protein levels in COAD patients.

C. Sankey diagram exhibiting the sample distributions between NMF groups and TCGA COAD molecular classifications.

The median-lines in each box represented median value, different asterisks indicated the results of *p-value* (**P* < 0.05; ***P* < 0.01; ****P* < 0.001).


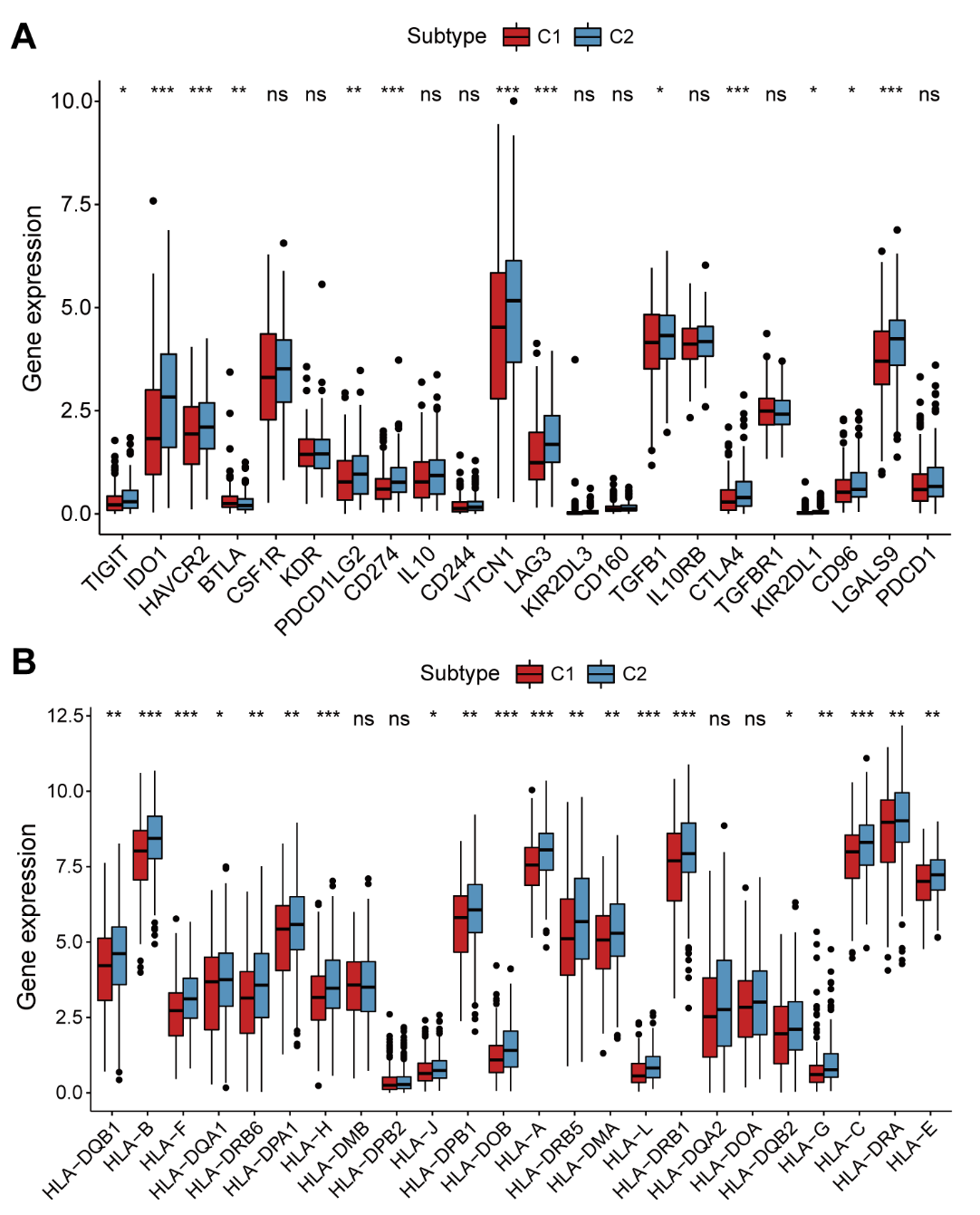


**Figure S13. Analysis on different biological features between different NMF subgroups in OV patients**

A. Boxplot showing the levels of immune checkpoint genes in OV patients.

B. Boxplot showing the levels of HLA proteins in OV patients.

The median-lines in each box represented median value, different asterisks indicated the results of *p-value* (**P* < 0.05; ***P* < 0.01; ****P* < 0.001).


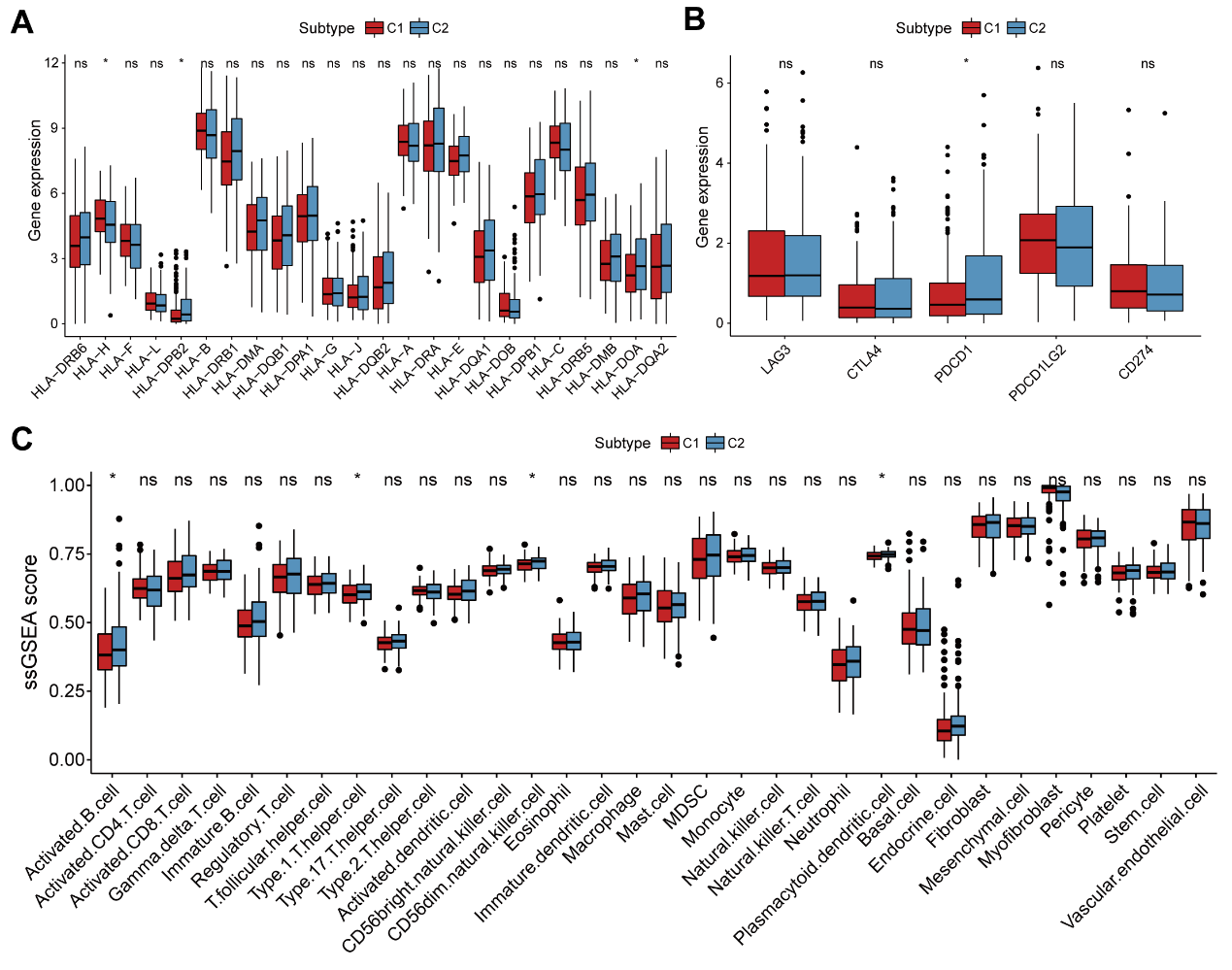


**Figure S14. Analysis on different biological features between different NMF subgroups in SARC patients**

A. Boxplot showing the levels of HLA proteins in SARC patients.

B. Boxplot showing the levels of immune checkpoint genes in SARC patients.

C. Boxplot showing the infiltrations of immune and stromal cells in SARC patients. The median-lines in each box represented median value, different asterisks indicated the results of *p-value* (**P* < 0.05).
