# Supplementary material for: Pan-cancer patterns of cuproptosis markers reveal biologically and clinically relevant cancer subtypes
Source: Biomark Res. 2023 Jan 31;11:13. doi: 10.1186/s40364-022-00446-5 (PMC9887831; doi:10.1186/s40364-022-00446-5)
Supplement: Supplementary file 3 — Additional file 3 . [file 40364_2022_446_MOESM3_ESM.docx]

**Supplementary Methods**

**Collection of cuproptosis regulators**

The information on 10 cuproptosis-associated genes (CuAGs) was collected from the article by Tsvetkov *et al* [1]. Those CuAGs encode the components of the lipoic acid pathway (FDX1, LIAS, LIPT1, DLD) and the pyruvate dehydrogenase complex (DLAT, PDHA1, PDHB, MTF1, GLS, CDKN2A). All those genes were mapped with Ensemble IDs.

**Data acquisition and preprocessing**

The multi-omics data were obtained from The Cancer Genome Atlas (TCGA) database, including transcriptome data, mutation data, copy number variation (CNV) data, and clinical data. We investigated 21 cancer types with relatively high morbidity and mortality. The expression data were downloaded with Fragments Per Kilobase of transcript per Million mapped reads (FPKM) and were log-transformed. Whole genomic somatic mutation data were downloaded from the GDC TCGA project on the UCSC Xena platform [2], and the single nucleotide variant (SNV) data were further masked by the Varscan algorithm. Clinical data including overall survival (OS), progression-free survival (PFS), disease-free survival (DFS), age, gender, and other clinical parameters were downloaded from the cBioportal research network [3].

**Gene Set Cancer Analysis (GSCA)**

GSCA is a multi-functional cancer analyzing platform with multi-omics data from TCGA and GETx databases [4]. We used GSCA to summarize the SNV and CNV status of the CuAGs in different cancers. The drug-targeted identification of the CuAGs was estimated based on the Genomics of Drug Sensitivity in Cancer (GDSC) database. The Spearman correlations between CuAGs expressions with IC50 of distinct therapeutic drugs were calculated.

**Expression analysis of cuproptosis genes**

Ten cancer types with corresponding normal tissues were selected for differential gene expression (DGE) analysis. We compared the gene expression data of cancer and normal tissues by the Wilcox test to identify the differentially expressed CuAGs. The survival R package was employed for univariate cox proportional hazards regression to evaluate the prognostic relevance of the CuAGs in each cancer. Additionally, we randomly selected 100 samples from each cancer and employed the Pheatmap R package to exhibit the expression of the CuAGs. Also, we calculated the immune and stromal scores of each sample by the Estimate R package and performed Pearson’s correlation analysis to investigate the correlations between CuAGs and biological features (i.e., TMB, MSI, immune and stromal scores).

**NMF algorithm for unsupervised clustering**

We used the NMF R package to classify samples based on the expression patterns of the 10 CuAGs. The clustering number was set from 2 to 10, and we further determined the average profile width of the common member matrix. According to cophenetic, rss, and silhouette, the optimal number of clusters was evaluated. Rtsne R package was further employed to validate the clustering results and visualized the multidimensional data. Additionally, we compared the multi-omics parameters among subgroups by ANOVA. The Survival R package was employed to compare the overall survival difference among subgroups in each cancer.

**Biological features analysis**

Genes of 2 cuproptosis correlated pathways, Hypoxia pathway and Reactive oxygen species pathway, were obtained from the h.all.v7.5.1.symbols.gmt in The Molecular Signatures Database (MSigDB). The corresponding scores of those 2 pathways were calculated by the ssGSEA algorithm based on the GSVA R package.

To evaluate the TME of each sample, we applied GSVA to calculate the infiltration levels of innate and adaptive immune cells, and stromal-correlated cells such as activated B cells, Macrophages, and Fibroblasts. The activations of TME-related pathways, such as the EMT pathway, WNT target pathway, and Angiogenesis, were also calculated. Additionally, the DNA repair-related pathways were estimated for a better understanding of genomic stability. The representative cells and pathways for GSVA analysis were obtained from previous studies [5-8], which were listed in Additionally File 2: Table S7-S9. We compared the level of each component/pathway among subgroups by ANOVA, and results of *p-value* < 0.05 were considered significant.

We analyzed the data from the TCIA platform to investigate the immunotherapy response of immune checkpoint genes. With the immunophenoscore (IPS) obtained from each sample, we compared the predicted therapeutic effects of anti-PD1 and anti-CTLA4 between different subgroups.

**Statistical analysis**

All statistical analyses were performed using R version 4.1.2. Results of *p-value* < 0.05 were considered statistically significant.

**References:**

1. Tsvetkov P, Coy S, Petrova B, Dreishpoon M, Verma A, Abdusamad M, Rossen J, Joesch-Cohen L, Humeidi R, Spangler RD, et al. Copper induces cell death by targeting lipoylated TCA cycle proteins**.** *Science*. 2022;375**:**1254-1261. doi:10.1126/science.abf0529

2. Wang S, Xiong Y, Zhao L, Gu K, Li Y, Zhao F, Li J, Wang M, Wang H, Tao Z, et al. UCSCXenaShiny: An R/CRAN Package for Interactive Analysis of UCSC Xena Data**.** *Bioinformatics*. 2021. doi:10.1093/bioinformatics/btab561

3. Cerami E, Gao J, Dogrusoz U, Gross BE, Sumer SO, Aksoy BA, Jacobsen A, Byrne CJ, Heuer ML, Larsson E, et al. The cBio cancer genomics portal: an open platform for exploring multidimensional cancer genomics data**.** *Cancer Discov*. 2012;2**:**401-404. doi:10.1158/2159-8290.CD-12-0095

4. Liu CJ, Hu FF, Xia MX, Han L, Zhang Q, Guo AY. GSCALite: a web server for gene set cancer analysis**.** *Bioinformatics*. 2018;34**:**3771-3772. doi:10.1093/bioinformatics/bty411

5. Bindea G, Mlecnik B, Tosolini M, Kirilovsky A, Waldner M, Obenauf AC, Angell H, Fredriksen T, Lafontaine L, Berger A, et al. Spatiotemporal dynamics of intratumoral immune cells reveal the immune landscape in human cancer**.** *Immunity*. 2013;39**:**782-795. doi:10.1016/j.immuni.2013.10.003

6. Zhang X, Lan Y, Xu J, Quan F, Zhao E, Deng C, Luo T, Xu L, Liao G, Yan M, et al. CellMarker: a manually curated resource of cell markers in human and mouse**.** *Nucleic Acids Res*. 2019;47**:**D721-D728. doi:10.1093/nar/gky900

7. Mariathasan S, Turley SJ, Nickles D, Castiglioni A, Yuen K, Wang Y, Kadel EI, Koeppen H, Astarita JL, Cubas R, et al. TGFbeta attenuates tumour response to PD-L1 blockade by contributing to exclusion of T cells**.** *Nature*. 2018;554**:**544-548. doi:10.1038/nature25501

8. Cao R, Ma B, Wang G, Xiong Y, Tian Y, Yuan L. Characterization of hypoxia response patterns identified prognosis and immunotherapy response in bladder cancer**.** *Mol Ther Oncolytics*. 2021;22**:**277-293. doi:10.1016/j.omto.2021.06.011
